# Connectivity, Not Frequency, Determines the Fate of a Morpheme

**DOI:** 10.1371/journal.pone.0069945

**Published:** 2013-07-29

**Authors:** Daniela Barbara Keller, Jörg Schultz

**Affiliations:** Department of Bioinformatics, Biocenter, University of Würzburg, Würzburg, Germany; Universidad Carlos III de Madrid, Spain

## Abstract

Morphemes are the smallest meaningful parts of words and therefore represent a natural unit to study the evolution of words. To analyze the influence of language change on morphemes, we performed a large scale analysis of German and English vocabulary covering the last 200 years. Using a network approach from bioinformatics, we examined the historical dynamics of morphemes, the fixation of new morphemes and the emergence of words containing existing morphemes. We found that these processes are driven mainly by the number of different direct neighbors of a morpheme in words (connectivity, an equivalent to family size or type frequency) and not its frequency of usage (equivalent to token frequency). This contrasts words, whose survival is determined by their frequency of usage. We therefore identified features of morphemes which are not dictated by the statistical properties of words. As morphemes are also relevant for the mental representation of words, this result might enable establishing a link between an individual’s perception of language and historical language change.

## Introduction

Already Charles Darwin was aware of similarities between language change and biological evolution. In ’The Descent of Man’ he writes in 1871 ’The formation of different languages and of distinct species, and the proofs that both have been developed through a gradual process, are curiously parallel’ [1]. Accordingly, methods to delineate the history of languages have been interchanged with those used in the reconstruction of the phylogeny of species and vice versa [2]. But, parallels can be identified on further levels than language and species. Muller noted in 1870 that the most striking analogy is not the ‘struggle for life among separate languages’ but the ‘struggle for life among words and grammatical forms which is constantly going on in each language’ [3]. Thus, methods developed for the study of biological evolution might also be useful for the analysis of language change. The factors driving language change can be classified as internal and external ones [4]. The internal factors are the physical conditions, like the physiology of the human speech organs and psychological factors like perception, processing and learning of language. On the other hand, the external factors are for example expressive use, prestige and stigma, education and language contact. In the case of words it was shown quantitatively, that the frequency of usage determines their fate [5], [6]. But words are not the only unit to analyze language change even when focusing on vocabulary change. It happens only rarely that a so far meaningless string becomes associated with a meaning. This was the case for example for the English word ‘zilch’ which means ‘nothing’ [7]. More frequently, new words are borrowed from another language [8]. This process can be followed by a change of meaning. Arguably even more frequently new words arise by the fusion of two so far not related words or meanings. As an example, the word of the year 2010 in Germany was ‘Wutbürger’ (anger-citizen) denoting middle-class people who are increasingly unsatisfied with political decisions. It was generated by fusing two words (‘Wut’–anger and ‘Bürger’–citizen) [9]. Thus, to understand the evolution of words, one also has to look at the parts which compose a word. So called morphemes are the minimal meaning bearing units of words. As one word can be built by multiple morphemes, one morpheme can be found in different words. The study of how these morphemes can be combined to yield words is the central question of morphology [10]. In this descriptive structural linguistic view, morphemes are seen as discrete units which are combined to build words. There has been a longstanding debate whether this structure is also mirrored in the mental lexicon (the human word-store) and the processing of words. Today, most models assume complete storage for some words and (partial) de-composition for others [11], [12]. Variants of these hybrid models differ on which words are decomposed and how this decision is made. Still, they all agree in the explicit storage for at least some morphemes. Contrasting models do not represent morphemes as discrete entities in the mental lexicon [13], [14]. These distributed connectionists approaches assume that ‘the same general principles that govern phonological and semantic processing of whole words and sentences govern the processing of the subparts of words commonly called morphemes’ [15]. This model could be rejected, if ‘there would be residual effects owing to morphological structure *per se*’ after ‘the statistical properties of words were equated’ [16] Here, we report on the identification of such residual effects by exploiting an analogy of words and proteins which enabled the application of an approach from bioinformatics. Usually, arguments in favor of one or the other model are drawn from psycholinguistic studies of well selected small sets of words. Contrasting, we performed a comparative historical analysis based on dictionary data and large word lists over time to investigate language change. As ‘[language] change is both a window into cognitive representations and a creator of linguistic patterns’ [17] we expected a reflection of the structure of the mental lexicon in language change.

## Materials and Methods

### Word Lists

Our analyses cover 200 years of English and German which are related, but slightly different in their degree of synthesis [18], i.e. German has more morphemes per word than English. As we were mainly interested in derivational word-formation, ‘the relationship between lexemes of a word family’ [10], we deliberately omitted inflection (different word forms of a lexem) by using dictionaries and lemmatized word lists. We defined a word as a head entry in a dictionary or as the lemma of the lemmatized corpora. Possible blank characters within a word like in ‘window pane’ were used as morpheme boundaries. The following dictionaries and corpora were used: Johnson – English 18^th^ century [19], Webster – English beginning 20^th^ century [20], BNCbaby – English end 20^th^ century [21], Adelung – German 18^th^ century [19] and WDG – German 20^th^ century [22].

### Morpheme Detection

Morphemes were identified automatically by Morfessor version 1.0 [23] with default settings. The decomposition into morphemes was evaluated for 18^th^ century German (Adelung) and 20^th^ century German (WDG), respectively, by comparing the results to a 1% sample of manually decomposed words. 84.37% of the decompositions in WDG were correctly identified with a false positive rate of 15.63% and a false negative rate of 36.15%. In Adelung 85.64% of decompositions were correct with a false positive rate of 14.36% and a false negative rate of 27.44%. In total, 83% of the morphemes in WDG and 86% of those in Adelung were correctly identified. Within the Morpho Challenge 2010, Morfessor 1.0 was evaluated on a gold standard set for English and German with a graph-based assignment algorithm. It reached a precision of 0.8686 and a recall of 0.7226 for English and a precision of 0.8128 and a recall of 0.4806 for German [24].

### Morpheme Networks

**Figure 1:**
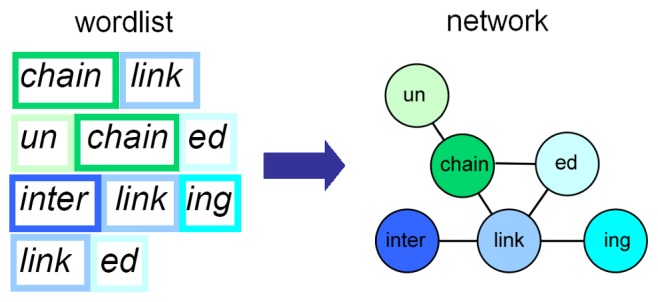
Generating a morpheme network out of a word list. Each morpheme is a node and is connected to other morphemes if they can be found next to each other in at least one word.

For analyzing the morphemes and their relationships, we used an approach which was successfully applied to the analysis of proteins and domains, the structural, functional and evolutionary units of proteins [25]. Like a morpheme in words, one domain can be found in different proteins and one protein can harbor many domains. We used this analogy to build morpheme networks. Here, morphemes are nodes which are connected if they can be found next to each other in at least one word, see Figure 1. Thus, our focus is on formatives, which ‘recur in the morphological analysis of word-forms’ independent of whether or not they are also morphemes [26]. This fits well to the algorithm implemented by Morfessor 1.0, which searches for the optimal concise set of units such that every word in the data can be formed by concatenation of some units [27]. For ease of understanding nevertheless the term morpheme is used in the following.

A network was built for each word list with morphemes as nodes and an undirected edge between morphemes if they occur side by side in a word. Thus, when analyzing the word ‘beautifulness’, no edge between ‘beauti’ and ‘ness’ would be drawn, as these are no direct neighbors. Analyses with directed edges (according to reading order) gave similar results. Multiple and loop edges were skipped. Network analyses, calculations and graphics were performed in R version 2.14.2 [28]. To describe the characteristics of the network, different measures were calculated based on the topological properties of the nodes. As overall measures the size (number of edges and number of nodes), the mean connectivity (mean number of edges per node), the mean path length (mean shortest connection between every two nodes) and the mean clustering coefficient were calculated. The clustering coefficient of a node describes the likeliness of two neighbors of this node to be connected to each other [29]. The mean clustering coefficient of the network is the mean of the clustering coefficients of all nodes. A small mean path length L ∼ ln(N) with N is the number of nodes reveals the small-world property [29]. If the mean path length is even smaller with L ∼ ln(ln(N)), the network is called ‘ultra-small’ [30]. Looking at the connectivity distribution P(k) reveals the scale-free property if P(k) ∼ k^−γ^ and thus follows a power law [31]. Another feature of the network is the hierarchical organization which can be identified by the dependence of the clustering coefficient from the connectivity of the nodes C(k) ∼ k^−a^ [32]. The assortativity value of a node is the average connectivity of its neighbors. The dependency between assortativity and connectivity shows assortative or disassortative mixing of the network [33] which was confirmed by calculation of the Spearman correlation. A positive dependency would show assortative mixing where nodes with high connectivity tend to be linked to again highly connected nodes. Disassortative mixing, proven by a negative relationship between connectivity and assortativity, would show that highly connected nodes tend to link to poorly connected ones.

### Word Frequency

To investigate the relation of morpheme properties to word use, the frequency of the 250.000 most frequent lemmata from DeReKo [34] was used for todays German and the frequency of the lemmata in the BNC corpus [35] for todays English. Confidence intervals for the difference between the frequency of usage of new and old words were calculated. Frequency of usage was transformed with base-2 logarithm according to the definition of frequency class in linguistics [36]. This measure is calculated in relation to the most frequent word in the corpus, which is assigned to the frequency class zero. A word that is approximately half as frequent belongs to the frequency class one. If a word has the frequency class n, this means that the most common word is 2^n^ times more frequent.

### Fixation of Morphemes

To delineate different factors influencing the fixation of morphemes we used logistic regression models with the factors frequency (defined as the sum of the frequency of all words containing a morpheme) and the connectivity (the number of different direct neighbors of a morpheme in the analyzed words). Transformation (natural logarithm and base 2 logarithm) and standardization were applied on connectivity and frequency to ensure comparability of the results. To exclude effects of multicollinearity, single factor models were performed additionally.

### Emergence of New Connections

To investigate the relationship of the number of new connections of a morpheme to its connectivity in both networks and its frequency of usage, bivariate and partial correlations were calculated and compared. Again logarithmically transformed values were used.

## Results

### Morpheme Networks Reveal Language Dynamics

As a tool to study language change, we created networks for word list covering 200 years of English and German (see Material and Methods). In these networks the morphemes are represented as nodes and an undirected edge is drawn between two morphemes occurring next to each other in a word. We defined the number of neighbors of a morpheme as its connectivity.

**Figure 2:**
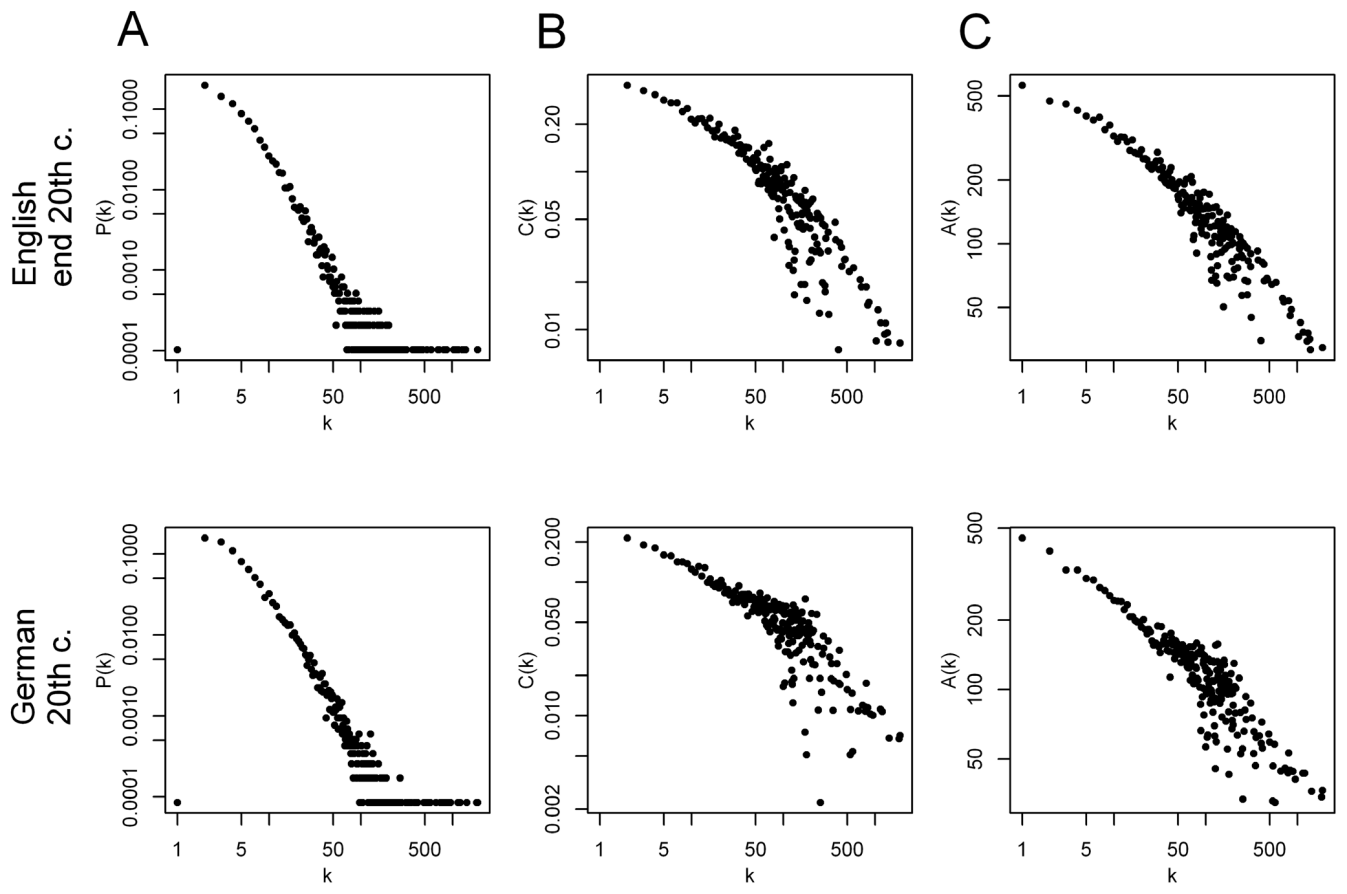
Global network properties of two networks as an example. Remaining networks show similar properties. **A** Scale-free: connectivity distribution follows a power law except for very small k. **B** hierarchical: clustering coefficient decreases with increasing connectivity. **C** Disassortative: negative correlation between neighbor’s connectivity and connectivity.

Considering the global architecture, all morpheme networks showed the same topological features, i.e. they were ultra-small, scale-free (except for very small k), hierarchical and disassortative (Figure 2 and Table 1). A key feature of scale-free networks is the existence of a small number of nodes with an exceptionally large number of neighbors, called hubs. These hub-morphemes are present in many different words. As expected, the largest hubs (the morphemes with the most direct neighbors) are affixes like ‘un’ and ‘ly’ in English and ‘en’ and ‘ver’ in German. In contrast to affixes, base morphemes are those morphemes which can also be found as stand-alone words. If base morphemes are hubs, they should represent concepts important for the specific time. Base morphemes emerging as hubs in all networks were for example ‘house/haus’, ‘water/wasser’ and ‘wood/holz’ indicating a common cultural background of these Germanic languages (rank values for all examples are listed in Table 2). The 18^th^ century networks are dominated by terms from nature like ‘wort’, ‘kraut’ (herb), ‘baum’ (tree) and ‘sea’. In contrast, in the 20^th^ century data work and leasure time related terms come up like ‘time/zeit’, ‘dienst’ (service), ‘spiel’ (game, play), ‘free’ and ‘life’. Thus, historical differences of hub-morphemes highlight cultural changes.

**Table 1:** Key values of the networks.

	English			German	
	18^th^	begin 20^th^	end 20^th^	18^th^	20^th^
	Johnson	Webster	BNCbaby	Adelung	WDG
n	37588	45236	63077	54663	86129
N	6547	7683	9544	7049	11256
E	33410	42932	55910	50675	77817
k	10.21	11.18	11.72	14.38	13.83
L	2.99	3.01	3.00	3.04	3.11
ln(N)	8.79	8.95	9.16	8.86	9.33
ln(ln(N))	2.17	2.19	2.22	2.18	2.23
C	0.21	0.22	0.24	0.18	0.15
r	−0.4403	−0.3785	−0.3531	−0.3494	−0.2866

n: number of entries in the word list; N and E: number of vertices and edges in the network, respectively; k: mean connectivity; L: mean path length; ln(): natural logarithm; C: mean clustering coefficient; r: assortativity calculated as the Spearman-correlation.

**Table 2:** Rank values according to connectivity within the network.

	German 18^th^ century		German 20^th^ century		English 18^th^ entury		English end 20^th^ entury	
	Morpheme	Rank	Morpheme	Rank	Morpheme	Rank	Morpheme	Rank
Affixes	en	1	en	2	Un	3	un	10
	ver	4	ver	5	Ly	5	ly	8
Common base morphemes	wasser (water)	15	wasser (water)	30	Water	199.5	water	148
	holz (wood)	18	holz (wood)	44	Wood	156.5	wood	102.5
	haus (house)	26	haus (house)	29	House	108.5	house	146
Terms from nature	baum (tree)	20	baum (tree)	82.5	Sea	88.5	sea	160
	kraut (herb)	23	kraut (herb)	549	Wort	95	wort	4987.5
Terms from work and leisure time	zeit (time)	89.5	zeit (time)	31	Time	298.5	time	152
	dienst (service)	159	dienst (service)	46	Life	1073	life	268
	spiel (game, play)	138.5	spiel (game, play)	50	Free	335	free	169

**Figure 3:**
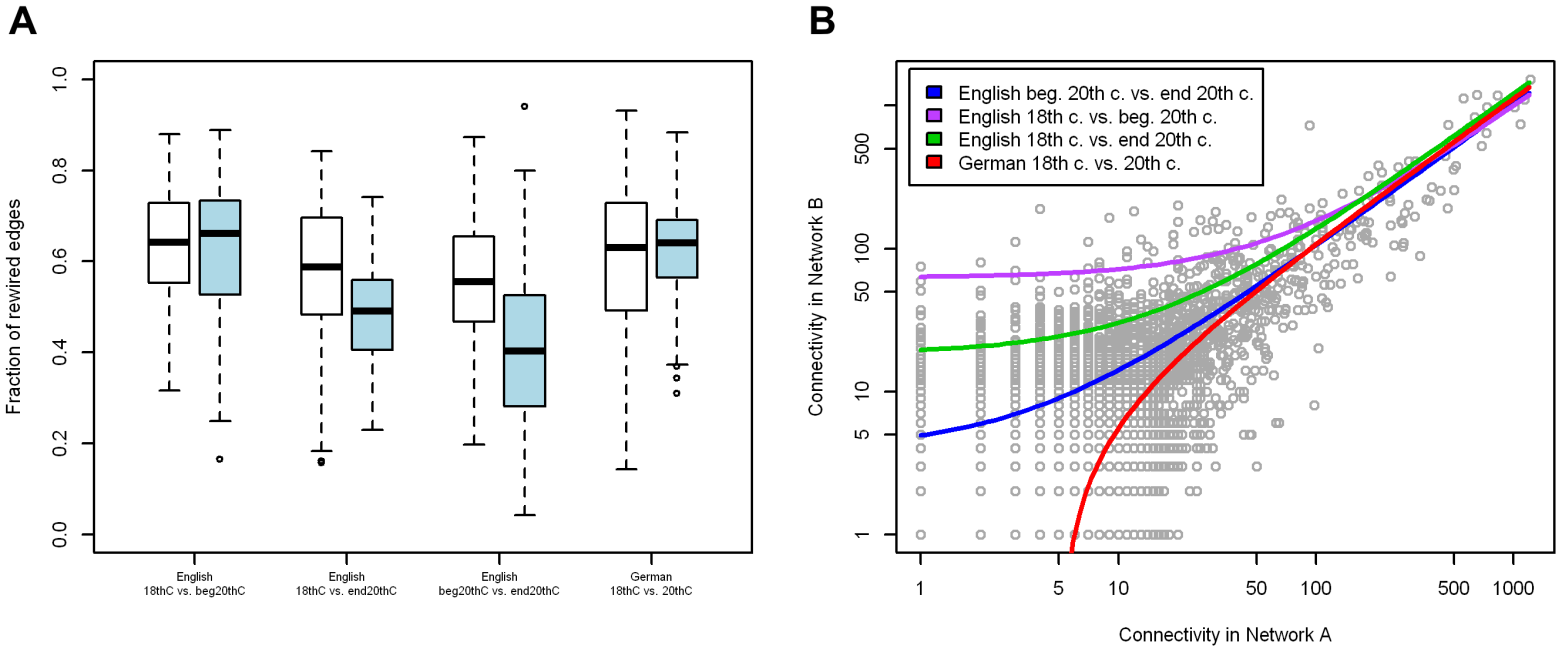
Connectivity of hub-morphemes. **A** Boxplots of fractions of re-wired edges of hub-morphemes (connectivity ≥50). Blue = lost edges, white = gained edges. **B** Hubs stay hubs – connectivity values of two compared networks (English beginning 20^th^ century vs. end 20^th^ century, grey dots). Lines correspond to the fitted linear models on hub-values for each comparison.

**Figure 4:**
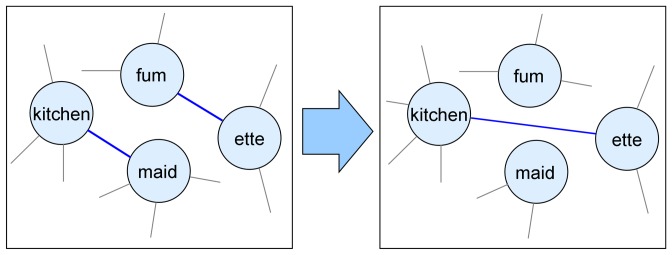
Example for rewiring from English 18^th^ to 20^th^ century. Whereas in the 18^th^ century ‘kitchenwork’ resulted in ‘fumette’, in the 20^th^ century one cooks in a ‘kitchenette’.

**Figure 5:**
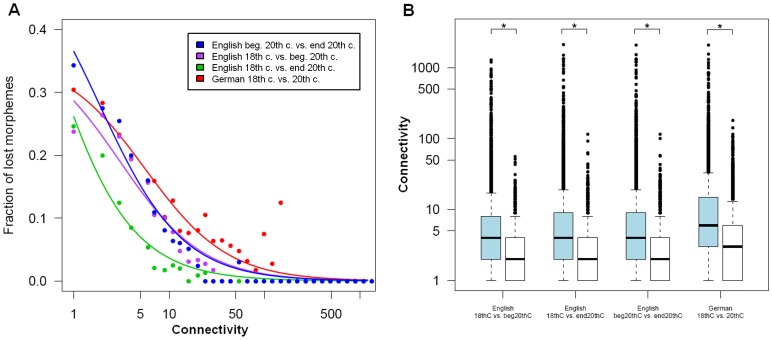
Connectivity versus loss and gain of morphemes. A Fraction of lost morphemes dependent on connectivity. Fit of the function y = a/(x+b) with least squares. **B** Comparison of connectivity of shared (blue) and gained (white) morphemes (* indicates p-value <0.001).

To identify trends in the emergence and loss of morpheme complexes and morphemes we mapped networks onto each other using identical morphemes as anchors. To minimize errors in the identification of cognate morphemes and effects of differing performance of decomposition, network comparisons were performed only within the languages. Thus, comparisons were made between German 18^th^ century and 20^th^ century, between English 18^th^ and beginning 20^th^ century and between English beginning 20^th^ century and end 20^th^ century. Even when considering only morphemes present in both networks, between 48% and 72% of the edges were changed (Table 3). This change is caused on the one hand by the loss of all words containing two specific morphemes as neighbors. On the other it is due to the invention of new direct combinations of existing morphemes. Although highly connected morphemes changed many of their connections (Figure 3 A), they stayed highly connected (Figure 3 B). Together, this reveals that the re-wiring of existing morphemes like in the example in Figure 4 is a major mechanism in word formation. But also loss and gain of morphemes has an important influence. Between 10% and 43% of the morphemes were gained or lost over time within one language (Table 3). Typically, poorly connected morphemes were the most probable candidates to get lost and gained morphemes were sparsely linked (Figure 5). Still, there are exceptions. The morphemes ‘zeidel’ (beekeeping term) and ‘seiger’ (miner’s term for vertical layers) were lost from the 18^th^ to the 20^th^ century German although they were highly connected. Inversely, the morphemes ‘auto’ (car), ‘industrie’ (industry) and ‘film’ (movie) were not present in words of the 18^th^ century, but are highly connected in the 20^th^ century. These exceptional cases can home in on morphemes which invaded a language in a short time-span.

**Table 3:** Percentage of interchanged nodes and edges comparing networks in time.

Comparison		Morphemes		Edges	
		loss	gain	Loss	gain
English	18^th^ vs. beg20^th^	19.78	27.25	53.84	64.13
	beg20^th^ vs. end20^th^	21.59	32.50	71.07	74.94
	18^th^ vs. end20^th^	14.31	33.31	60.85	73.52
German	18^th^ vs. 20^th^	20.45	45.00	67.08	73.12

### Connectivity Influences Morpheme Fixation

We showed that the morphemes’ connectivity influences their survival. But there are other factors like the frequency of usage of a morpheme (how many times do words containing a specific morpheme occur) which could also be of importance. To delineate the factors influencing the fixation of morphemes, we used logistic regression models.

The logistic regression model predicted whether a morpheme is new or was already present in the previous point in time by the factors connectivity in the recent network and frequency (German: R^2^ discrimination index = 0.170, English: R^2^ discrimination index = 0.129). In the English model both factors were significant, but the coefficient of connectivity was larger than that of frequency. In the German model only the connectivity had a significant influence in addition to a large coefficient. The coefficient of frequency was nearly zero and not significant (Table 4). The single factor models with factor connectivity showed a much better fit than the models with the single factor frequency (Table 4). Thus, connectivity is a more important factor behind the fixation of new morphemes than frequency.

**Table 4:** Results of logistic regression models.

	Two factor model			Single factor models			
	Connectivity		Frequency	Connectivity		Frequency	
	R^2^	Coefficient (p)	Coefficient (p)	R^2^	Coefficient (p)	R^2^	Coefficient (p)
English	0.129	0.6845 (<0.0001)	0.4604 (<0.0001)	0.108	0.8689 (<0.0001)	0.077	0.7291 (<0.0001)
German	0.170	0.8497 (<0.0001)	−0.0080 (0.7728)	0.170	0.8451 (<0.0001)	0.057	0.4395 (<0.0001)

p: p-Value.

### The Number of New Connections Correlates to Connectivity

Our result of the network analyses showed that the change of connections - the re-wiring of morphemes – is an important component of word formation. Applying partial correlation, we next investigated the relationship of the amount of new connections to the old and recent connectivity of a morpheme and its frequency of usage. Pairwise correlations show high positive values for the number of new connections and connectivity in the recent network (Table 5). Moderate positive correlations were identified between the number of new connections with connectivity in the older network and with the frequency. Comparing these values to the partial correlation delineated the true relationships without confounding variables. The partial correlation without frequency showed only slight difference to the pairwise values indicating that frequency has a low correlation to the number of new connections. Partial correlations corrected for two factors finally showed that the connectivity of the recent network has the strongest relationship to the number of new connections (German 0.9275, English 0.9331). Thus a high number of new connections relates to a high connectivity in the recent network, which is not surprising. This is followed by a negative relationship between the number of new connections and the connectivity in the older network (German −0.4326, English −0.5029). Thus highly connected morphemes in the older network build only few new connections, whereas low connected morphemes will acquire more connections. In contrast, the partial correlation coefficient of frequency and the number of new connections is nearly zero (German −0.0727, English −0.0623) showing that there is no influence from the frequency of usage on the number of new connections.

**Table 5:** Pairwise and partial correlation coefficients for the relation to the number of new connections.

	English			German		
related variable	pairwise	partial withoutfrequency	partial without bothother variables	pairwise	partial without frequency	partial without both other variables
connectivity in older network	0.4894	0.4916	−**0.5029**	0.4436	0.3957	−**0.4326**
connectivity in recent network	0.9410	0.9321	**0.9331**	0.9499	0.9247	**0.9275**
frequency	0.3854	/	−**0.0623**	0.5790	/	−**0.0727**

## Discussion

Network approaches are not new in language studies. This includes different levels ranging from for examples networks of interconnected words, syntactic networks and semantic networks. These networks have been used to observe and explain universal of languages [37]. Also in cognitive science language networks are used for investigating neural networks and cognitive processes, ‘shedding new light on how knowledge is stored and exploited’ [38]. These networks are typically composed of interconnected words. With looking at the connections between morphemes within words we extend the network approach to a new subject. In our implementation the network is undirected and unweighted. Obviously, this is a strong abstraction. Still, we decided to omit directionality, as left-to-right order might imply a directionality which when looking at semantics would be better represented by a hierarchy. Similarly, we did not consider the number of co-occurrences of two morphemes (which one could interpret as weights) as we were mainly interested in the formation of new words.

As a first result of our approach we found that cultural changes are reflected in changes of hub morphemes, i.e. the morphemes with the highest type frequency. This obviously does not come as a surprise. Still, it is a new approach to study ‘culturomics’. So far, these studies counted the occurrence of specific words (lemmatized or not) over a given time, i.e. they worked with token frequencies [39]. Challenges of this approach are first the large number of words and second that related words associated with the same concept are independent. Basing the analysis on type instead of token frequency might enable circumventing these challenges. First, the number of morphemes is vastly smaller than that of words. For example the WDG with 86,129 words is broken down to 11,256 different morphemes. Second, focusing on morphemes enables to group related words together. Furthermore, the analysis is on a more abstract level and might therefore enable the identification of higher level trends. Admittedly, the meaning of one morpheme can differ between two words and thus noise is added to the analysis. Still we suggest that morphemes are a well suited level to study the interaction between cultural and language change.

With the analysis of morpheme networks of English and German over 200 years, we identified connectivity as a major factor behind morpheme and word evolution. But, how does ‘connectivity’ relate to existing linguistic terms? Connectivity counts the number of morphemes which are direct neighbors to a given morpheme in all analyzed words. This differs, albeit slightly, from the type frequency, which counts all words containing a given morpheme. The difference can be illustrated with the words ‘beautiful’ and ‘beautifulness’. Here, ‘beauti’ has only one direct neighbor, ‘full’. Thus the connectivity is one. Contrasting, the type frequency of ‘beauti’ would be two. Still, connectivity and type frequency are highly correlated and therefore the first can be seen as a proxy for the latter. Thus we showed that type frequency influences the evolution of morphemes.

In the case of words, token frequency has a strong influence [5], [6]. The token frequency is defined as the number of occurrences of a word in a given corpus, e.g. how many times the word ‘beautifulness’ can be counted in a given text. The influence of token frequency holds true also on the comparably small time-scale analyzed here. The 95% confidence intervals for the difference in means of the frequency class values of old (existing in both data sets) and new words (existing only in the newer data set) range from 2.32 to 2.40 for English and from 2.34 to 2.46 for German. Hence both confidence intervals lie completely in the range of the frequency class 2. Therefore old words in both English and German are used four times (2^2^) more frequent than new words.

Contrasting words, it is the type and not the token frequency which determines the fixation as well as the death rate of morphemes. This outcome was unexpected, especially when assuming an utterance based model of language change [40]. In the case of morphemes it seems to be more important for the survival that it is used in many different combinations than how many times it is used. Thus, it is still a kind of usage that defines the evolution, but one has to carefully check what the key factor of usage is.

Furthermore, the connectivity is not only the key factor behind survival and death of morphemes, it also correlates with the productivity of a morpheme. A morpheme or a linguistic pattern in general is called productive if new words are build based on the morpheme or pattern. There have been many different definitions of productivity and different approaches to measure it [41]. If one sees the connectivity as a proxy for the type frequency, the amount of new connections can be interpreted as the productivity of a morpheme (arguably a most basic approach). Again, it was the type and not the token frequency which correlates with the productivity of a morpheme. Surprisingly, when following 200 years of language change, the correlation was negative, i.e. a morpheme with a high type frequency has a lower productivity than one with a lower type frequency. This means, that counter-intuitively one seems to avoid too frequent morphemes when building new words.

In summary, word-formation patterns are not only created by the statistics of words but indicate a morphological structure. We conclude that, in a historical view, morphemes are discrete units with features which cannot be explained by the statistics of words alone. This finding, although based on dictionaries and word lists, can directly be related to models of the mental lexicon [42]. Within the framework of the distributed connectionist model of the mental lexicon such word independent features cannot be expected. Rather, our results give additional evidence for the discrete mental representation of morphemes.

From another view, our results re-call studies from psycholinguistics. In the case of words, the recognition accuracy and response time in word/non-word classification depends on token frequency [43]–[45]. But when looking at morphemes, the response time is not determined by the token frequency of the morpheme or of the words containing it. Instead the family size, which can be equaled to the type frequency and thereby connectivity, is a significant predictor [46]–[48]. Thus, the same regularities were identified behind an individual’s processing of language (psycholinguistics) and historical language change (this study). Therefore, with this exploratory study we gave quantitative evidence for the importance of language processing as an internal factor for historical language change.
